# Predicting the macrovascular contribution to resting-state fMRI functional connectivity at 3 Tesla: A model-informed approach

**DOI:** 10.1162/imag_a_00315

**Published:** 2024-10-15

**Authors:** Xiaole Z. Zhong, Jonathan R. Polimeni, J. Jean Chen

**Affiliations:** Rotman Research Institute at Baycrest, Toronto, ON, Canada; Department of Medical Biophysics, University of Toronto, Toronto, ON, Canada; Athinoula A. Martinos centre for Biomedical Imaging, Massachusetts General Hospital, Charlestown, MA, United States; Department of Radiology, Harvard Medical School, Boston, MA, United States; Harvard-MIT Program in Health Sciences and Technology, Massachusetts Institute of Technology, Cambridge, MA, United States; Department of Biomedical Engineering, University of Toronto, Toronto, ON, Canada

**Keywords:** blood-oxygenation-level-dependent fMRI, vascular bias correction, resting-state fluctuation amplitude (RSFA), functional connectivity, vascular anatomical network (VAN), biophysical modelling

## Abstract

Macrovascular biases have been a long-standing challenge for functional magneticresonance imaging (fMRI), limiting its ability to detect spatially specificneural activity. Recent experimental studies, including our own, foundsubstantial resting-state macrovascular blood-oxygenation level-dependent (BOLD)fMRI contributions from large veins and arteries, extending into theperivascular tissue at 3 T and 7 T. The objective of this study is todemonstrate the feasibility of predicting, using a biophysical model, theexperimental resting-state BOLD fluctuation amplitude (RSFA) and associatedfunctional connectivity (FC) values at 3 Tesla. We investigated the feasibilityof both 2D and 3D infinite-cylinder Models as well as macrovascular anatomicalnetworks (macro-VANs) derived from angiograms. Our results demonstrate that (1)with the availability of macro-VANs, it is feasible to model macrovascular BOLDFC using both the macro-VAN-based model and 3D infinite-cylinder Models, thoughthe former performed better; (2) biophysical modelling can accurately predictthe BOLD pair-wise correlation near to large veins (with R^2^rangingfrom 0.53 to 0.93 across different subjects), but not near to large arteries;(3) compared with FC, biophysical modelling provided less accurate predictionsfor RSFA; (4) modelling of perivascular BOLD connectivity was feasible at closedistances from veins (with R^2^ranging from 0.08 to 0.57), but notarteries, with performance deteriorating with increasing distance. While ourcurrent study demonstrates the feasibility of simulating macrovascular BOLD inthe resting state, our methodology may also apply to understanding task-basedBOLD. Furthermore, these results suggest the possibility of correcting formacrovascular bias in resting-state fMRI and other types of fMRI usingbiophysical modelling based on vascular anatomy.

## 1 Introduction

The use of resting-state blood-oxygenation level-dependent (BOLD) functional magneticresonance imaging (rs-fMRI) has become increasingly valuable in assessing brainhealth and mapping brain connectivity (Srivastava etal., 2022;van den Heuvel &Hulshoff Pol, 2010). There has been extensive use of this technique inthe study of numerous neurological conditions (Ovadia-Caro et al., 2014;Vemuri et al.,2012), as well as in the study of brain development and ageing processes(Andrews-Hanna et al., 2007;Geerligs et al., 2015). The most common methodfor calculating functional connectivity (FC) using resting-state magnetic resonanceimaging (rs-fcMRI) is Pearson’s correlation analysis (Bandettini et al., 1993;Biswal et al., 1995), in which the BOLD signals from seed voxels arecorrelated with those from the rest of the brain. Commonly, correlation isinterpreted as reflecting synchronous neuronal activity (Biswal et al., 1995;Panet al., 2015). The contribution of the macrovasculature to functionalconnectivity (FC) in the grey matter (GM) has previously been demonstrated in humandata (Huck et al., 2023;X. Z. Zhong et al., 2024). Specifically, ourinvestigation indicated that, at 3 Tesla, such effects occurred not only within theconfines of macrovasculature but also more than 10 mm away from individualmicrovascular blood vessels, consistent with findings by others (Bernier et al., 2021;Huck et al., 2023) including at 7 T. However, there is still limitedsystematic investigation into macrovascular contributions to rs-fMRI.

Unlike in rs-fMRI, the question of “vein versus brain” has been arecognized challenge for many years in task-based fMRI.Boxerman et al. (1995)demonstrated, through both rodentexperiments and simulation studies, that macrovasculature affectsgradient-recalled-echo (GRE) BOLD contrast more than microvasculature. Despite thefact that microvascular sensitivity increases with an increase in the main magneticfield (Menon & Goodyear, 1999), veinsstill account for the majority of the contribution to GRE BOLD signals at 7T (Menon, 2012;UIudag et al., 2009) and show strong association with both BOLD signalamplitude (1.5T) and timing (3T) (Provencher et al.,2018;Vigneau-Roy et al., 2014).Previous studies in rats showed that venous structure could be detected directlyusing fMRI (Hyde & Li, 2014), and thatthe BOLD response from veins is nearly double that from microvessels in brain tissueat 11.7 T (Yu et al., 2012). Moreover,several studies have suggested that penetrating arteries and ascending veins also donot strictly reflect local neural activity (Uludağ & Blinder, 2018), despite their much smaller sizerelative to the macrovasculature investigated to date.

Numerous approaches have been developed to remove the venous BOLD effect. Thesimplest method of correcting the bias appears to be to mask out the detectablemacrovasculature using vascular masks, such as segmented from angiograms. However,as work by ourselves and others have shown (Bernieret al., 2021;Huck et al., 2023;X. Z. Zhong et al., 2024), macrovasculareffects can extend well into the perivascular tissue, making masking-basedcorrection inadequate. Spin-echo (SE) BOLD has also been proposed as an alternativeto the conventional GRE BOLD to suppress the strong macrovascular susceptibilityeffects. Nevertheless, this option sacrifices signal-to-noise ratio (Menon, 2012), and the use of typical echo-planar imaging(EPI) readouts even with SE refocusing is still susceptible to large-vein T2*effects (Goense & Logothetis, 2006;Ragot & Chen, 2019). The BOLDimage phase has also been proposed as a regressor to remove macrovascular effects intask-based fMRI (Menon, 2002;Stanley et al., 2021), assuming that the signal magnitudeand phase scale with one another, and that the intravascular (IV) contribution isdwarfed by the extravascular (EV) contribution due to low voxel resolution atconventional field strengths. Phase regression has even been shown to be effectiveat reducing large-vein contributions even at high field and high resolution (Stanley et al., 2021). However, extraction ofaccurate phase values can be a challenge. As vascular diameter increases or as thevoxel size decreases, it becomes less appropriate to ignore the IV phase offsets andvascular orientation effects, as discussed in our previous work (X. Zhong & Chen, 2022,^2023^). Most recently,Huck et al. (2023)advocated the use of higher order polynomials formodelling the fall-off of extravascular fields over space and subsequently removingvenous biases, both proximal and distal to the vasculature. However, the authorsconcluded that their model is not sufficient to correct venous bias in rs-fMRI data.In light of these recent findings, it may be necessary to consider a morecomprehensive modelling approach for the vascular BOLD contributions as the initialstep in correcting for them.

The modelling of macrovascular contributions to GM FC with a closed-form analyticalmodel is a challenging undertaking. Earlier work byOgawa, Menon, et al. (1993)demonstrated that the BOLD signal isdependent on the orientation and diameter of the vessels, as well as bloodoxygenation, but some of these parameters are difficult to measure reliably in thehuman brain. The dependence of the BOLD signal on vascular geometry (mainly of largepial veins) was also evident in the biophysical simulations based on realisticvascular anatomy (Gagnon et al., 2015;X. Zhong & Chen, 2022) and in in-vivofMRI measurements (Fracasso et al., 2018;Viessmann et al., 2019). Furthersimulations also revealed that the position of a large vessel position within anfMRI voxel also plays a significant role in modulating macrovascular BOLD (Sedlacik et al., 2007;X. Zhong & Chen, 2023), increasing the complexityof modelling macrovascular effects. Despite not having been investigated previously,other factors (such as patient positioning and spatial resolution) may also have asignificant impact on modelling macrovascular effects.

Instead of using a closed-form analytical model, it is possible to use a numericalmodel for calculating macrovascular BOLD signals that may simplify these issues. Akernel-based approach, proposed in previous studies (Cheng et al., 2009;Salomir et al.,2003), involves convolving kernels (representing point-spread functions)with susceptibility maps to compute local field offsets and, consequently, togenerate a BOLD signal based on the blood susceptibility. The approach allows us todirectly simulate the BOLD signal arising from realistic macrovascular anatomicalnetworks (macro-VANs: macrovascular-specific human VAN), although with thelimitation that blood oxygenation remains unknown. It is important to note, however,that this numerical approach will result in approximation errors and require asignificant amount of computational resources (as will be discussed in latersections). In this regard, it remains to be seen whether the numerical approach withrealistic vasculature has an advantage over the analytical approach based on acylinder model.

The current study is intended to provide a detailed analysis of the macrovascularcontribution to FC using biophysical modelling. Moreover, two analytical models willbe tested in addition to a numerical model in terms of establishing the feasibilityof a theoretically driven framework for predicting the macrovascular BOLD signal inthe resting state. Furthermore, the investigation would be extended to theperivascular tissue in order to gain a better understanding of the macrovascularcontribution beyond the confines of the macrovasculature, which is of practicalimpact for connectivity mapping. As of the time of writing, this is the first studyto investigate the prediction of the macrovascular contribution to resting-state FCusing biophysical modelling.

## 2 Methods

### 2.1 Data set

In this work, we compare experimental rs-fMRI data with simulated rs-fMRI data ona voxel-wise basis. We used data selected from the Midnight Scan Club (MSC) dataset (Gordon et al., 2017). The studyprotocol was approved by the Human Studies Committee and Institute Review Boardat Washington University School of Medicine in accordance with the Declarationof Helsinki. These data can be obtained from the OpenNeuro database, withaccession number ds000224. Due to the high computational demand of our modellingapproach, we balanced computational cost with the generalizability of theresults, selecting data from four healthy right-handed young participants, twomales and two females, ages 28–34 years.

### 2.2 MRI acquisition

Each subject underwent 12 imaging sessions on a Siemens TRIO 3T MRI scanner(Siemens Healthcare GmbH, Erlangen, Germany) on separate days. Here, only theprotocols related to this study are listed.

In total, 12 of 2D time-of-flight (TOF) angiograms were acquired, including 4ascending (transverse, 0.6 × 0.6 × 1.0 mm^3^, 44 slices,TR = 25 ms, TE = 3.34 ms, flip angle(α)= 20^o^), 4 left-right encoded (sagittal, 0.8 × 0.8× 2.0 mm^3^thickness, 120 slices, TR = 27 ms, TE= 7.05 ms,α= 60^o^), and 4 anterior-posterior encoded (coronal, 0.7× 0.7 × 2.5 mm^3^thickness, 128 slices, TR = 28ms, TE = 7.18 ms,α= 60 degrees) data sets. Resting-state fMRI data were acquired with agradient-recalled-echo BOLD-weighted EPI sequence (TR = 2.2 s, TE= 27 ms,α= 90^o^, 4 mm isotropic resolution, 36 slices, scan time= 30 min). T1-weighted anatomical MPRAGE data were included as well(sagittal, 224 slices, 0.8 mm isotropic resolution, TE = 3.74 ms, TR= 2,400 ms, TI = 1,000 ms,α= 8 degrees). The participants were instructed to fixate on a whitecrosshair on a black background during the rs-fMRI scans. An EyeLink 1000eye-tracking system (SR-Research, Ottawa, Canada,http://www.sr-research.com)was used to monitor participants to determine whether they had any prolonged eyeclosures, which may indicate sleepiness. There was only one participant whoshowed prolonged eye closures during the scans.

### 2.3 fMRI processing and analysis

A summary of the rs-fMRI processing procedures can be found inFigure 1a. fMRI preprocessing pipeline wasimplemented with tools from FSL (Jenkinson etal., 2012), AFNI (Cox, 1996),and FreeSurfer (Fischl, 2012). Thefollowing steps were included in the preprocessing steps: (a) 3D motioncorrection (FSL MCFLIRT), (b) slice-timing correction (FSL slicetimer), (c)brain extraction (FSL bet2 and FreeSurfer mri_watershed), (d) rigid bodycoregistration of functional data to the individual T1 image (FSL FLIRT), (e)regression of the mean signals from white-matter (WM) and cerebrospinal fluid(CSF) regions (fsl_glm), (f) bandpass filtering to obtain frequency band0.01–0.1 Hz (AFNI 3dBandpass), and (g) spatial smoothing with a6 mm full-width half-maximum (FWHM) Gaussian kernel (FSL fslmaths). Foreach participant, after preprocessing, we calculated the voxel-wisevenous–venous, arterial–arterial, and arterial–venous FCmetrics based on the maximum cross-correlation coefficients for the voxels withpositive correlation coefficients and the minimum cross-correlation coefficientsfor the voxels with negative correlation coefficients. In spite of the fact thatmost fMRI connectivity analysis is based on Pearson’s correlations, weused cross-correlations in order to compare the theoretically predictedcorrelation coefficients directly with the experimentally measured ones withouttaking into account time lags.

**Fig. 1.**
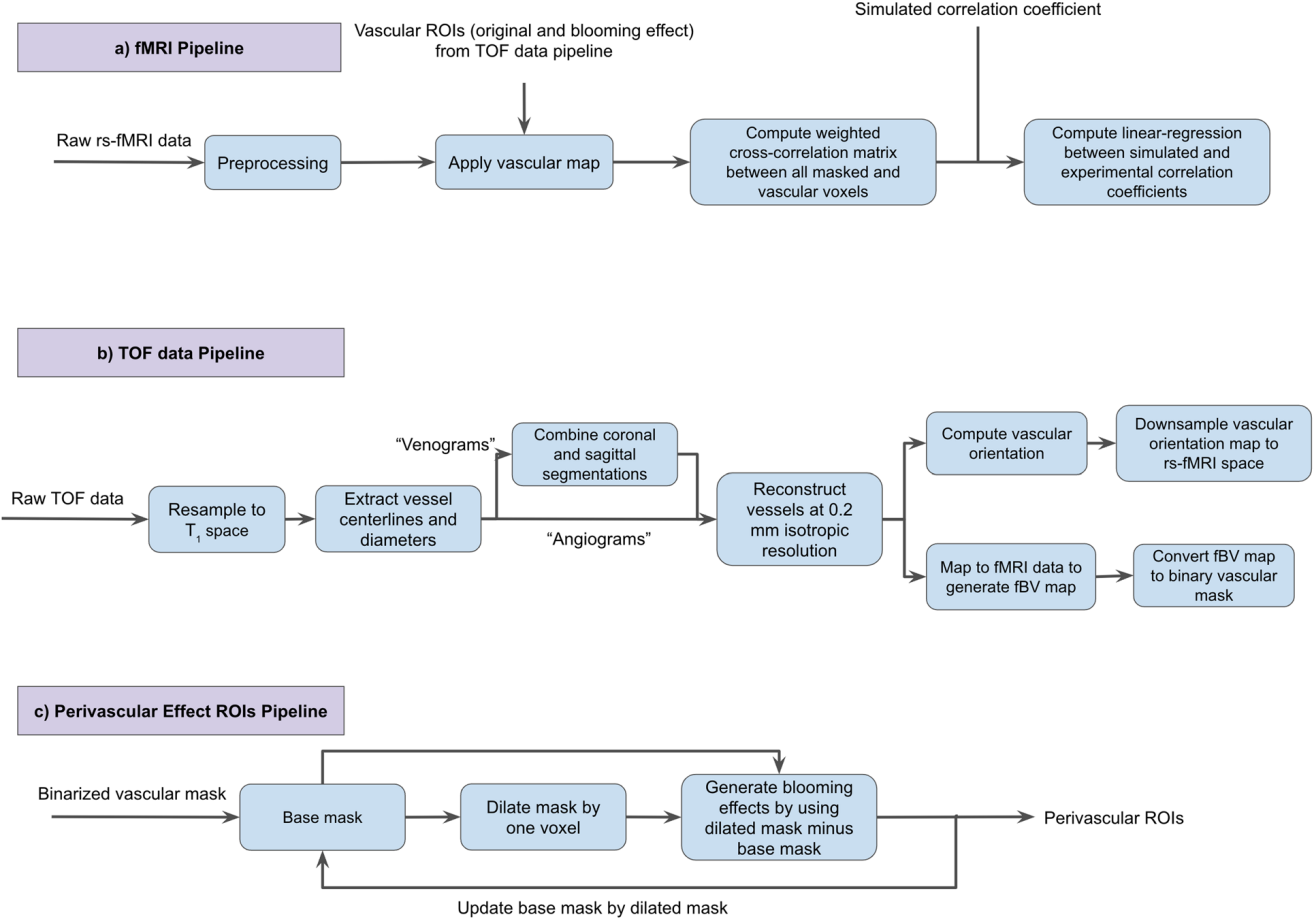
Overview of in-vivo data analysis procedure. (a) The fMRI data analysispipeline. (b) The TOF data preprocessing and segmentation pipeline. (c)The analysis pipeline for assessing the extravascular effects on rs-fMRImetrics.

### 2.4 Macrovascular segmentation and data processing

**Fig. 2.**
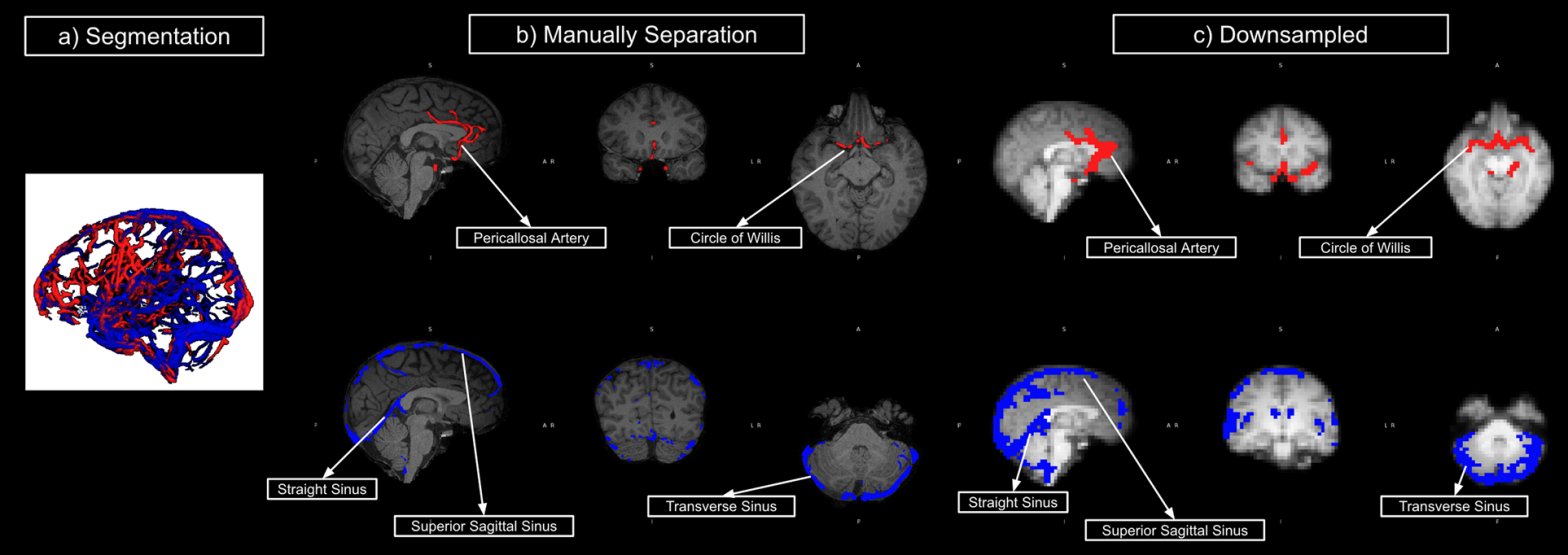
Illustration of macrovascular segmentation and processing. (a) Segmentedmacrovasculature. (b) Manually separated arteries and veins (asexamples, arteries: the pericallosal artery and Circle of Willis; veins:the straight sinus, superior sagittal sinus and transverse sinus). (c)Arterial and venous masks downsampled to fMRI resolution.

The strategies for macrovasculature segmentation and processing are summarized inFigure 1b. TOF images were registeredto T1 space (FSL MCFLIRT) and segmented using the Braincharter Toolbox (https://github.com/braincharter/vasculature) (Bernier et al., 2018) (Fig. 2a). Visual inspection was performed to ensure the absence ofmajor artefacts. The centreline image encoded with diameter information(“centredia” output from the Braincharter Toolbox) obtained fromthe raw images (at 0.8 mm isotropic voxel resolution) was pooled from all TOFimages across all encoding directions, and where vessel overlaps were detected,the highest diameter estimate is assumed for the overlapping vessel.Accordingly, the vessels used in the subsequent analysis are primarily majorcerebral vessels with a minimum diameter of close to 0.8 mm (e.g., the superiorsagittal sinus and the Circle of Willis). This combinatorial approach maximizedthe completeness and signal-to-noise ratio of the resulting macro-VANs. Thecombined centrelines were then upsampled to a resolution of 0.2 mm using AFNI(Cox, 1996) with nearest-neighbourinterpolation (3dresample). Based on each vascular centreline, a line wasconstructed that connects two vascular voxels that both neighbour the centralvoxel in a 9 × 9 × 9 voxel matrix. Vascular orientation wascalculated as the angle between this line and the z-axis (zenith) and x-axis(azimuth). The zenith angle is further corrected by applying position metrics inMRI header files to compensate for the difference between theparticipants’ slice direction and the B_0_direction (scannerz-axis).

Vascular blood-volume fraction (fBV) and orientation maps were manuallyregistered to BOLD space using coordinates from AFNI’s volume selectionfunction (3dAutobox). fBV was estimated by counting the number ofhigh-resolution voxels occupied by vessels in each fMRI voxel (4 mm isotropicvoxel resolution), and macrovascular maps were derived from binarized fBV maps.In order to ensure the accuracy of registration, necessary quality controlmeasures have been added. Due to the fact that all TOF images contained botharteries and veins, each arterial and venous map was manually separated based onan anatomical atlas (Tortora &Derrickson, 2018) (Fig. 2b, c).Lastly, the measured voxel-wise values of orientation and fBV are used to createdifferent simulated voxels containing blood vessels, described in thefollowing.

### 2.5 Simulations

**Fig. 3.**
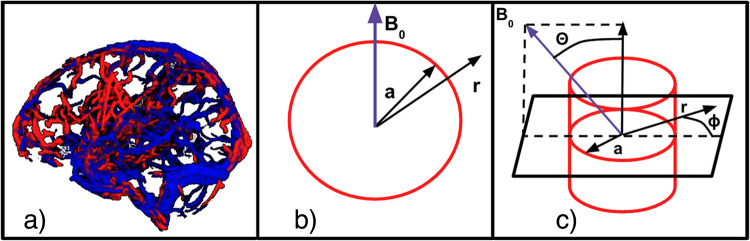
Illustration of three vascular models used to predict the rs-fMRI BOLDsignal. (a) vascular-based macro-VAN simulation (macro-VAN Model). (b)The two-dimensional (2D) infinite-cylinder model (2D Cylinder Model).(c) The three-dimensional (3D) infinite-cylinder model (3D CylinderModel). Here,*a*is the cylinder radius,*r*is the distance between the point of interest andthe centre of the cylinder cross-section, B_0_is the mainmagnetic field, θ is the angle between the B_0_and thecylinder axis, and φ is the angle between the vector<**r**> and the projection of B_0_on the plane perpendicular to the cylinder axis.

All simulation parameters and the values used in all models (Fig. 3) are listed inTable1. In the remainder of this work, the tabulated parameter values areassumed unless otherwise stated. We do not expect the results to be affected bythe choice of specific*Y*for arteries, veins, and tissues. Asthe spatial extents of B_0_inhomogeneities generated by large vesselsare much greater than the diffusivity of water molecules at body temperature(Jensen et al., 2005), we assumednegligible diffusion effects. The simulated BOLD scan parameter values (e.g., TRand TE) were adjusted to match the parameter values used in the rs-fMRI scans.At these settings, the arterial inflow effect is deemed negligible (Gao et al., 1996). The*Y*-dependent T_2_of tissue and blood was calculatedaccording to previous relaxometry studies (UIudag et al., 2009).

**Table 1.** Simulation parameters and values.

Parameter	Definition	Simulated value	Source
∆χ	Susceptibility of blood with fully deoxygenated blood	4 x π x 0.27 x 10 ^-6^	( Spees et al., 2001 )
Hct	Hematocrit	0.4	Men: 40-54%; Women: 36-48% ( Billett, 1990 )
Voxel size	N/A	4 mm isotropic	According to in-vivo rs-fMRI acquisition protocol ( Gordon et al., 2017 )
TR	Repetition time	2.2 s	
TE	Echo time	27 ms	
α	Flip angle	90 deg	
B _0_	Main magnetic field	3T	
Y _a_	Arterial oxygenation level	0.98	( Leeper, 2015 )
Y _v_	Venous oxygenation level	0.6	( Fan et al., 2014 )
Y _tissue_	Tissue oxygenation level	0.85	( Gagnon et al., 2015 )
T1 _blood_	T1 of blood	1,649 ms	( Zhang et al., 2013 )
T1 _tissue_	T1 of tissue	1,465 ms	( Shin et al., 2009 )

For all three simulation models, these values were set to defaultunless otherwise stated.

**Table 2.** Summary of all resolutions used in simulation.

Sub-voxel Resolution for macro-VAN (used for macro-VAN Model simulation)	40 μm isotropic
Upsampled Resolution (used for reconstructing the macrovasculature for macro-VAN Model simulation, is subsequently broken into sub-voxels, seen above)	0.2 mm isotropic
T1 Resolution	0.8 mm isotropic
rs-fMRI Resolution	4 mm isotropic

A brief summary of each resolution is also provided.

#### 2.5.1 Macro-VAN model

**Fig. 4.**
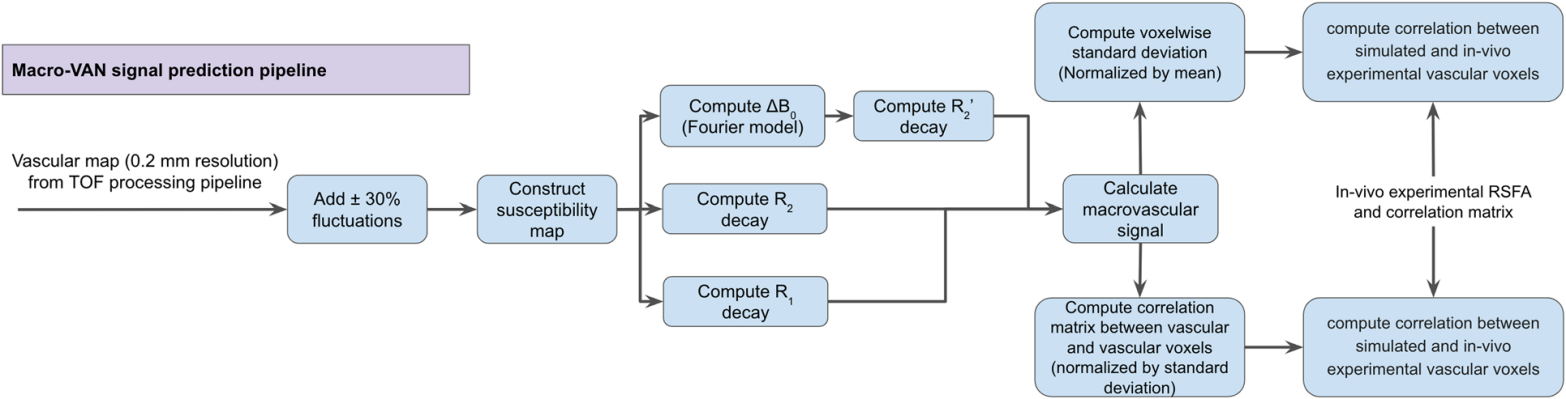
Overview of the simulation procedure (based on vascularsegmentation).

In this model, magnetic susceptibility maps are based on experimentallyacquired macro-VANs (Fig. 4), based onthis acquisition, all resolutions used in the simulation pipeline are listedinTable 2. A mask of thevasculature was generated by upsampling the macro-VANs to 0.2 mm isotropicresolution (The upsampling aims to allow at least four voxels to define theminimum vascular diameter (~0.8 mm, based on the spatial resolution of theTOF images), in order to minimize the ringing artefact due toundersampling), and necessary zero-padding by the size of a full field ofview on each side of the matrix to avoid cycle convolution wraparound. Theexperimental data and simulated data were first aligned with the coordinatesextracted from the processed macrovascular segmentation, as describedearlier. To minimize computational complexity, the susceptibility map wasderived using a Fourier-based model-independent convolution approach (Fig. 4) (Eqs. 1and2) (Cheng et al., 2009;Salomir et al., 2003), asfollows.

$$\Delta B_{z}={\text{FT}}^{-1}\left[\left(\frac{1}{3}-\frac{{\text{k}}_{\text{z}}^{\;2}}{{\text{k}}^{2}}\right)\text{FT}(\chi )\right]$$ (1)

χ=Δχ⋅Hct⋅(1−Y). (2)

FT denotes the Fourier transform, χ the local susceptibility,*k*_z_the distance in the k space along thez-axis, and*k*the distance in k-space($k^{2}=k_{x}^{2}+k_{y}^{2}+k_{z}^{2}$).R_2_’ is then calculated through the magnitude of thecomplex-valued mean magnetization of the dephasing spins resulting from theB_0_offset.

We further assumed the tissue type outside macrovasculature was grey matter(GM) to calculate the appropriate susceptibility difference between bloodand tissue. The mean BOLD signal was calculated as defined byEquations 3and4as

$$S_{T_{2\prime }}=\left|\mu \left(\text{exp}\left(i\gamma \Delta B_{z}\text{TE}\right)\right)\;\right|$$ (3)

S=sin(α)(1−exp(−TR /T1))/(1−cos(α)exp(−TR /T1)exp(−TE /T2))ST2′ (4)

whereγis the gyromagnetic ratio, and the operator|μ(·)|represents the magnitude of the mean of a complex number.

#### 2.5.2 2D cylinder model: 2D infinite cylinder

**Fig. 5.**
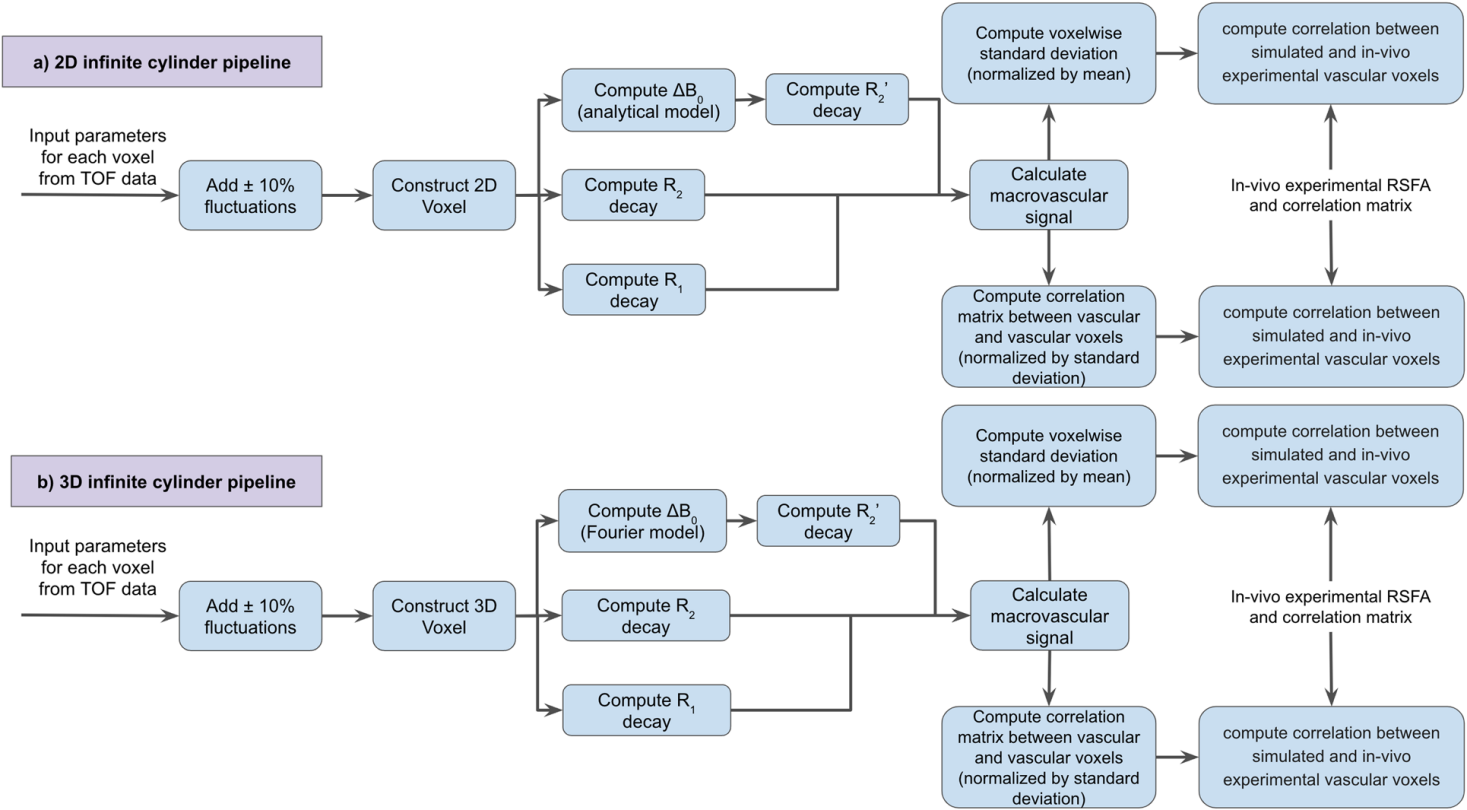
Overview of the simulation procedures for Models 2 and 3(infinite-cylinder based). To predict the rs-fMRI signal, threemodels were used, namely (a) the two-dimensional (2D)infinite-cylinder model, (b) the three-dimensional (3D)infinite-cylinder model. Correlations were computed for simulatedand experimental values of both RSFA and FC. The simulationparameters were extracted from the TOF data.

On the other end of the complexity spectrum is the single-voxeltwo-dimensional (2D) infinite-cylinder model, in which field offset isestimated in an analytical approach (Fig.5a) (Eqs. 5and6) (Ogawa, Menon, et al., 1993). Here, instead ofgenerating a volumetric mask of the macro-VAN, we calculated at eachlocation of the macro-VAN the diameter and orientation of the best-fittingcylinder and used this information (combined with the blood-tissuesusceptibility difference) to analytically calculate the magnetic fieldoffset. This calculation is based on a closed-form expression for theextravascular and intravascular field offsets for an infinite cylinder thatare a function of the vessel geometry and blood susceptibility, that is,

$$\Delta B_{z,\text{EV}}=2\pi \cdot \frac{\Delta \chi }{4\pi }\cdot \text{Hct}\cdot \left(Y_{\text{tissue}}-Y\right)B_{0}{\text{sin}}^{2}\left(\theta \right){\left(\frac{a}{r}\right)}^{2}\text{cos}\left(2\phi \right)$$ (5)

$$\Delta B_{z,\text{IV}}=\frac{2\pi \cdot \frac{\Delta \chi }{4\pi }\cdot \text{Hct}\cdot \left(Y_{\text{tissue}}-Y\right)B_{0}\left(3{\text{cos}}^{2}(\theta )-1\right)}{3},$$ (6)

where ω_0_is the main magnetic field in terms of angularfrequency; ∆χ, magnetic susceptibility of fully deoxygenatedblood; Hct, Hematocrit;*Y*, the percent blood oxygenation(*Y*_a_for artery and*Y*_v_for vein);(*Y_tissue_*−*Y*), thedegree of deoxygenation difference between blood and tissue; θ, theangle between the B_0_and the vascular axis;*a*,the cylinder radius;*r*, the distance between the point ofinterest and the centre of vascular cross-section; φ, the anglebetween vector <**r**> and B_0_projectionin the plane. This model assumes that the shape of the blood vessel is aninfinite cylinder with zenith θ, azimuth φ, and radius*a*derived from fBV. These input parameters can all beobtained from the arterial and venous segmentations based on the TOF data.This 2D Cylinder Mode is suitable when the magnetic susceptibility does notvary along the length of the cylinder, and facilitates our understanding ofthe dependence of IV and EV rs-fMRI signals on a single large vessel. As inthe case in the work ofBandettini and Wong(1995), we constructed a 2D voxel for each vascularorientation-fBV combination derived from the experimental data, divided into4,000 × 4,000 1-μm isotropic sub-voxels, with each voxeldefined by a set of vascular (arterial or venous) orientation and fBV valuesderived from the TOF data in the previous step. The 2D model is moreconducive to analytical calculations of susceptibility, and thedimensionality reduction can be traded off for higher spatialresolution.

#### 2.5.3 3D cylinder model: 3D infinite cylinder

This was a 3D version of the infinite-cylinder model, with the exception thatthe vessel is not assumed to penetrate the voxel at a right angle, such thatfBV will vary with θ, and that the susceptibility pattern can varyalong the length of the vessel. We constructed a 3D voxel containing 100× 100 × 100 40-μm isotropic sub-voxels, in which theinfinite cylinder is defined by the collection of measured parameters as wasdone in the 2D Cylinder Model. The field-offset estimation procedure is thesame numerical Fourier-transform-based method as used in the macro-VANModel. This model serves as an intermediate between the macro-VAN Model andthe 2D Cylinder Model, and is evaluated as a less computationally intensiveapproximation of the macro-VAN Model. The 2D and 3D Cylinder Models are bothsingle voxel and thus do not take into account interactions betweenneighbouring vessels that may influence EV field offsets.

#### 2.5.4 Temporal dynamics of the simulated vasculature

Vascular dynamics were simulated as sinusoidal variations at 0.1 Hz (Chu et al., 2018) for arterial fBV andvenous*Y*(Uludağ& Blinder, 2018;Vazquezet al., 2013). We used a sinusoid with ±10% variations(peak-to-peak) for Y and fBV fluctuations in the 2D and 3D Cylinder Models.For macro-VAN Model, the macro-VAN was upsampled to 0.2 mm isotropicresolution, and the smallest detectable vessels had approximate diameters of0.8 mm. Due to the coarser sample, the circular cross-section of each 0.8 mmvessel, assuming it is at the centre of its voxel, would be upsampled in 2Dinto a 13-voxel neighbourhood of 0.2-mm isotropic voxels. Thus, it wasnecessary to expand the fBV variations to ±30% to allow the quantizedvascular fBV to fluctuate by one voxel in the diameter dimension. Thesimulated BOLD signal can be calculated asEquation 4.

From these simulated BOLD signals, we computed both resting-state fluctuationamplitudes (RSFA) and FC (i.e., the correlation between voxel time courses).For computing the RSFA, both the simulated and experimental BOLD signalswere normalized by their respective means, with outlier time points (definedas lying beyond 1.5 quartiles of the mean) removed. Simulated FC metricswere calculated for voxel-wise arterial–arterial,venous–venous, and arterial–venous correlations with thesimulated signals from all three models. As the simulated data are noiselessbut are to be compared with noisy experimental results, we added noise foradditional realism. Thus, as suggested byGolestani and Goodyear (2011), we used the temporal standarddeviations of simulated time courses as a surrogate for SNR; that is, withnoise being similar across voxels, a higher baseline signal fluctuationwould translate into a higher SNR. We then multiplied the cross-correlationcoefficients in the FC metrics with the σ for each simulated voxel toobtain SNR-independent FC measurements.

The simulations were conducted using our servers equipped with 14 cores ofIntel Xeon X5687 CPU (at 3.6 GHz) (Intel Corporation, Santa Clara, CA,United States) and 180 GB of memory running Red Hat Enterprise Linux Server7.7 (Red Hat Inc., Raleigh, NC, United States). A customized simulationscript was written in MatLab 2019b (MathWorks Inc., Natick, MA, UnitedStates).

#### 2.5.5 Perivascular signal contributions

This is done for the macro-VAN case only. Predicted and measured rs-fMRIsignals were compared in the perivascular tissue as well.Figure 1cillustrates the methods used toassess perivascular effects of the macrovasculature on the rs-fMRI signal,whereby the perivascular ROIs were generated as follows. First, the arterialand venous segmentations were dilated by one voxel in 3D. Second, theoriginal vascular ROIs were subtracted from the dilated vascular ROIs. Thisproduced the extravascular ROI that is centred at one voxel distance fromthe centre of the vasculature. This process was repeated for perivasculardistances of two and three voxels (4 mm isotropic voxels). As discussed inthe previous section, cross-correlation-based connectivity values werecalculated separately for BOLD signals in arterial and peri-arterial ROIs,and between venous and peri-venous ROIs. This was done for both simulatedand in-vivo experimental signals.

### 2.6 Agreement between predicted and measured correlation coefficients

Each simulated voxel results in the simulated macrovascular-based BOLD signalspecific to a vascular location in the experimental data, from which acorresponding experimental BOLD signal can be extracted. In this way, toquantify model prediction accuracy, the simulated RSFA and vascular-drivencorrelation coefficients (FC values) predicted by all three models wereregressed against the experimental cross-correlation coefficients (FC value).All vasculature-related correlation coefficients (simulated and experimental)were then binned. Given that there are more voxels for venous ROIs than arterialROIs, the bin sizes were adapted approximately proportionately, that is, 100 forarterial–arterial correlations, 1,000 for arterial–venouscorrelations, and 10,000 for venous–venous correlations (as there aremore venous voxels than arterial voxels), to ensure a similar number of fitteddata sets for all three categories. This was done separately for each data set.The R^2^values were calculated to assess the quality of the predictionin terms of the simulated FC values. For perivascular BOLD, a similar regressionprocedure was conducted for the perivascular FC metrics using the macro-VANModel. The correlations were grouped by distance from the edge of thevasculature (i.e., one, two, or three voxels away). Similarly, the proceduredescribed above was applied to assessing the predictability of BOLD signalfluctuation amplitudes.

## 3 Results

### 3.1 Theoretical predictions based on a 2D infinite cylinder

**Fig. 6.**
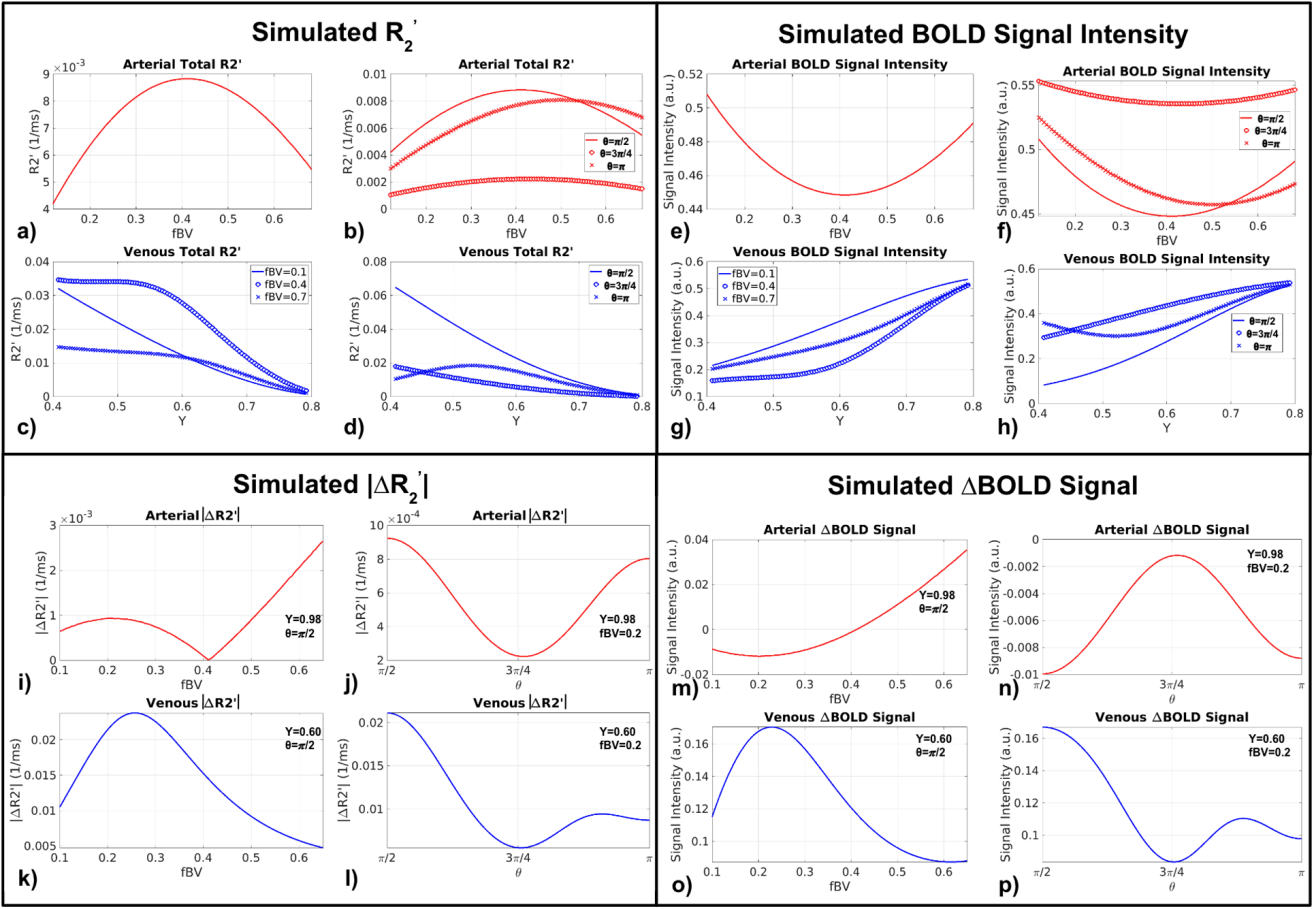
Influence of macrovascular fBV and*Y*onR_2_’ and BOLD signal intensity, simulated analyticallyusing the 2D Cylinder Model (a–h) and influence of macrovascularθ and fBV on resting-state R_2_’ fluctuations(|∆R_2_’|) and BOLD fluctuation(∆BOLD) (i–p). All simulations were performed using thedefault simulation parameters listed inTable 1and assuming 10% peak-to-peak oscillations in fBV(for arteries) or Y_v_(for veins).

The analytical equations (Eqs. 5and6) allowed us to gain anintuitive understanding of the interactions between the BOLD signal and variousvascular parameters. When simulated based entirely on the default parameterslisted inTable 1, arterialR_2_’ increased with fBV but only up to fBV = 0.4,and decreased as fBV increased further (Fig.6a). This is consistent with decreasing phase coherence as fBVincreases in a tissue-dominated voxel followed by an increasing phase coherenceonce the voxel becomes blood dominated. However, as the vascular orientationvaried, so did the behaviour of R_2_’ (Fig. 6b). The peak R_2_’ was generally notfound at the θ extrema (corresponding to when the vessel is parallel orperpendicular to B_0_). On the venous side, R_2_’declined with increasing Y_v_, but this behaviour also varied with fBV.Venous R_2_’ was maximized at an intermediate value of fBV. Asshown inFigure 6c, it was higher for fBV= 0.4 than for lower and higher fBVs (0.1 and 0.7, respectively). Lastly,for the default fBV, like arterial R_2_’, the venousR_2_’ dependence on Y_v_varied with θ, withR_2_’ higher at an intermediate value of 3π/4 than atθ of π/2 or π (Fig.6d). BOLD signal intensity was disproportionate to R_2_’(Fig. 6e–h): theR_2_’ was maximized at intermediate values of fBV, the baselineBOLD signal intensity was minimized at intermediate values of fBV; when theR_2_’ was maximized at intermediate theta values, thebaseline BOLD signal intensity minimized at intermediate theta values.

In rs-fMRI, fluctuations in R_2_’, rather than the meanR_2_’, is more relevant. The quantity∆R_2_’, computed as the difference between the maximumand minimum R_2_’ (associated with the maxima and minima in Yand fBV), can be viewed as the derivative of R_2_’ relative tothe oscillations in Y and fBV (Fig.6a–d). Arterial ∆R_2_’ initially rosewith increasing fBV, then decreased as fBV surpassed ~0.2, eventually increasingagain for fBV > 0.4 (Fig. 6i).Arterial ∆R_2_’ decreased with θ as θincreases to 3π/4 and then increased for θ up to π (minimumat θ = 3π/4) (Fig.6j). Venous ∆R_2_’ inFigure 6kshowed the opposite trend as arterial with fBV> 0.4 (Fig. 6i), which decreasedwith increasing fBVs. Lastly, like arterial |∆R_2_’|,venous |∆R_2_’| also decreased with θ untilθ = 3π/4, then increased for greater values of θ.Moreover, ∆R_2_’ became more stable while θapproached π. ∆BOLD was in turn computed as the difference in theBOLD signal corresponding to the two extremes of the input signals (fBV forartery and Y for vein), as derived from the associated R_2_’values. As seen inFigure 6m–p, themagnitude of ∆BOLD can be viewed as proportional to|∆R_2_’|, whereas the polarity of ∆BOLD willinfluence the correlation between ∆BOLD time courses. For instance, thevenous ∆BOLD inFigure 6oshowed theopposite trend as arterial ∆BOLD (Fig.6m), decreasing with increasing fBV until fBV ~0.2, increasing forhigher fBVs and remaining positive throughout all fBVs. Thus, an equivalentincrease in fBV for both an artery and a vein would lead to their BOLD timecourses remaining negatively correlated. Also, arterial and venous ∆BOLDshowed the same magnitude variation trend until arterial ∆BOLD changedpolarity at fBV ~0.4, which suggests a negative correlation between arterial andvenous BOLD time courses for fBV > 0.4. Lastly, arterial ∆BOLDincreased with θ until θ = 3π/4, then increased forgreater values of θ. This is contrary to the trend observed for venous∆BOLD (peaking at θ = 3π/4) (Fig. 6p).

### 3.2 Comparison between simulated and measured BOLD RSFA

**Fig. 7.**
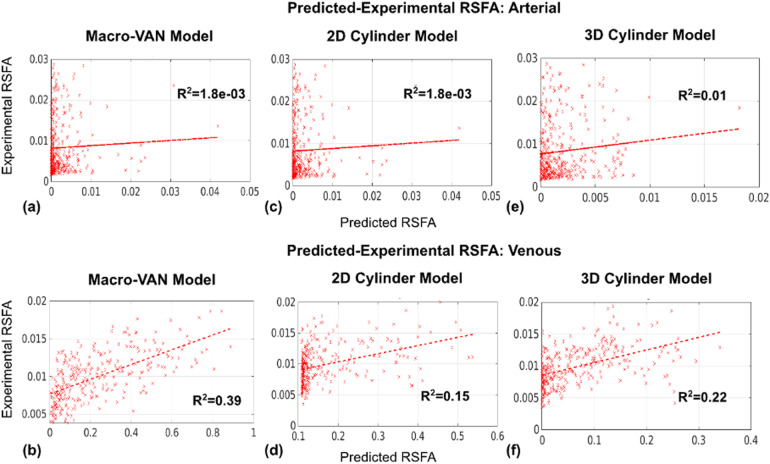
Regression between the in-vivo predicted and experimental vascular RSFAs.Regressions are shown between the in-vivo experimental and simulatedRSFA for macro-VAN Model (a, b), 2D Cylinder Model (c, d), and 3DCylinder Model (e, f). Data are from a representative subject. Each dotrepresents a bin average.

As shown inFigure 7a and dfor arepresentative data set, arterial RSFA is less well predicted than venous RSFAby the macro-VAN Model. For the 2D Cylinder Model, both arterial and venous RSFAare poorly predicted (Fig. 7c, d), possiblyattributable to a large cluster of measured BOLD RSFA values in voxels thatexhibit a low simulated RSFA. The 3D Cylinder Model is an improvement over the2D Cylinder Model especially for predicting venous RSFA, but the performanceremains lower than for the macro-VAN Model (Fig.7e, f). These trends are generalizable to all subjects (seeSupplementaryMaterials). SeeFigure S2for results for all subjects.

### 3.3 Comparison between predicted and measured correlation coefficients

**Fig. 8.**
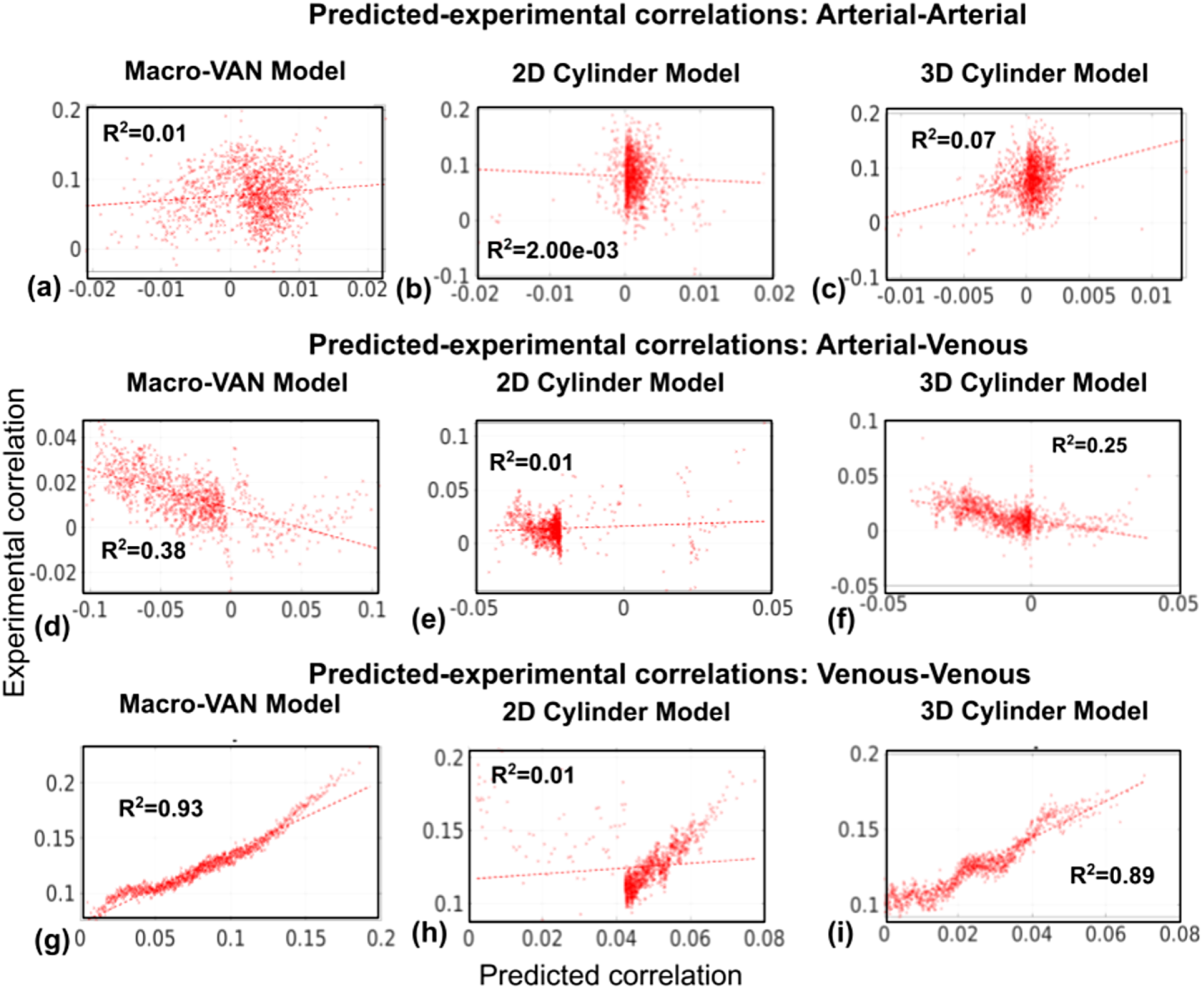
Regression between the in-vivo predicted and experimental vascularcorrelation coefficients. Data are from the same subject as shown inFigure 7. Regression lines forarterial–arterial correlations (a-c), arterial–venouscorrelation (d-f), and venous–venous correlation coefficients(g-i) are shown for all models. Each dot represents a bin average.

The simulated correlation coefficients based on the macro-VAN Model show thehighest predictiveness for venous–venous correlation coefficients (Fig. 8g), followed by arterial–venouscorrelation coefficients (Fig. 8d), andwith the lowest predictability for arterial–arterial correlationcoefficients (Fig. 8a). For the 2D CylinderModel, as expected, while significant positive associations between simulatedand in-vivo correlation coefficients dominated, the goodness of fit was low(Fig. 8b, e, h), with thearterial–arterial correlation R^2^being the lowest(<0.01). We also noted that the data points appear to sub-divide into twogroups, one spanning correlation coefficients of >0.04, and the other onespanning the area between 0 and 0.04. For the 3D Cylinder Model, thepredictability of venous–venous correlations remains remarkably high(Fig. 8i), followed byarterial–venous correlations (Fig.8f). The accuracy of the prediction of arterial–arterialcorrelations was relatively low (Fig. 8c),like in the cylinder models. SeeFigures S3–S5in Supplementary Materials forresults for all subjects.

### 3.4 Perivascular effects

#### 3.4.1 Perivascular contributions to GM BOLD signal standard deviations: predictivity using simulations

Only the macro-VAN provided the possibility to examine the perivasculareffects in a meaningful way, as it conveniently incorporates perivascularcontributions from neighbour vessels. We found the majority of vascularcontributions to the surrounding tissue to be limited to one voxel away (4mm isotropic) from the original vascular masks (Fig. S6inSupplementary Materials). As shown inFigure9, both arterial and venous perivascular RSFA are poorlypredicted by the macro-VAN Model, irrespective of the distance from thevascular voxels.

**Fig. 9.**
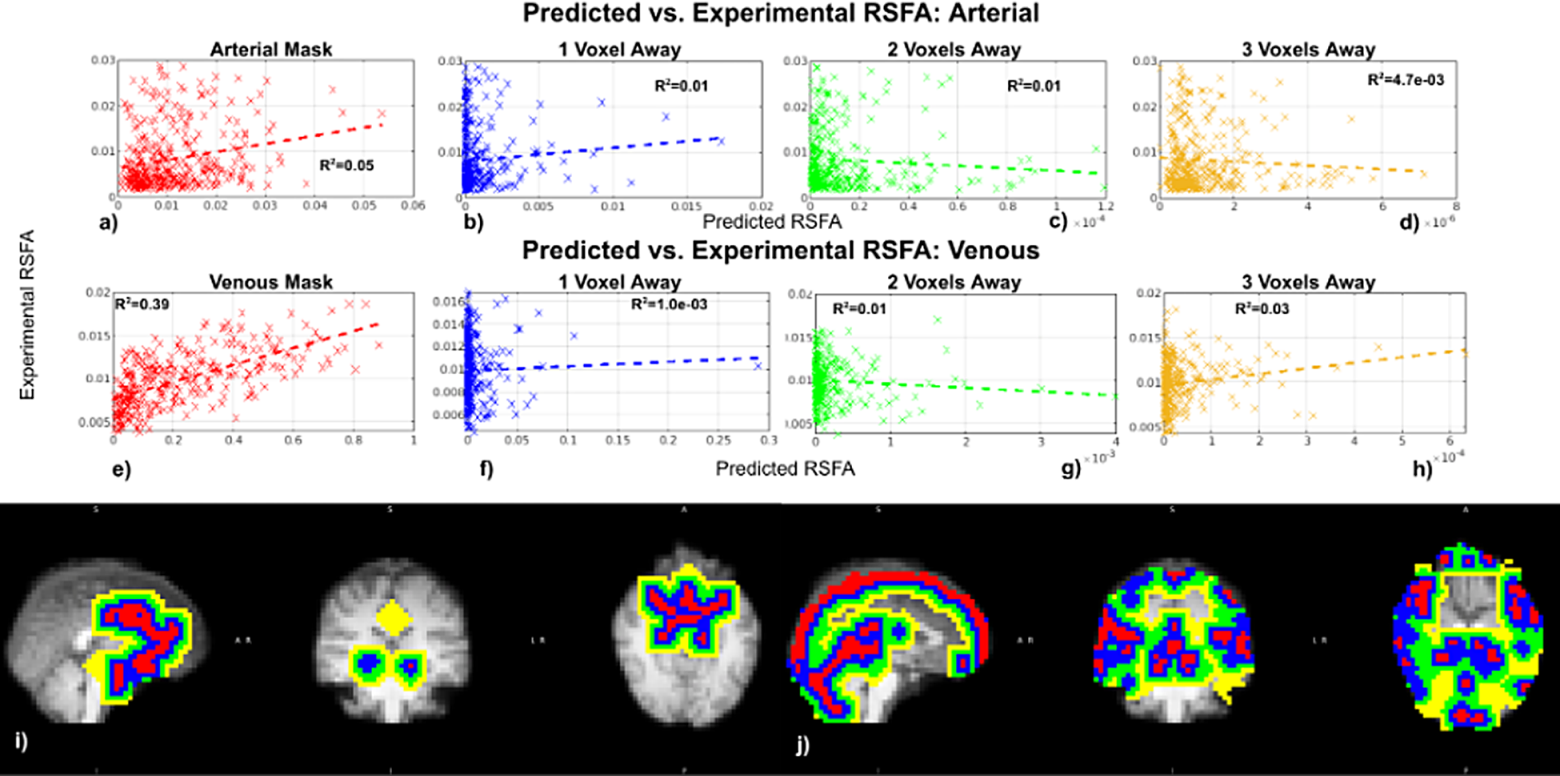
Dependence of perivascular BOLD standard deviation on distance fromvasculature: predicted versus experimental results (the macro-VANModel). Data taken from the same representative subject as shown inFigure 7. Perivascularmasks are shown for both arterial (a-d) and venous (e-h), withcolour coding as red—intravascular, blue—one voxelaway from the vascular voxel, green—two voxels away, andyellow—three voxels away (i, j). Each dot represents a binaverage.

#### 3.4.2 Perivascular contributions to GM BOLD signal correlations: predictiveness using simulations

**Fig. 10.**
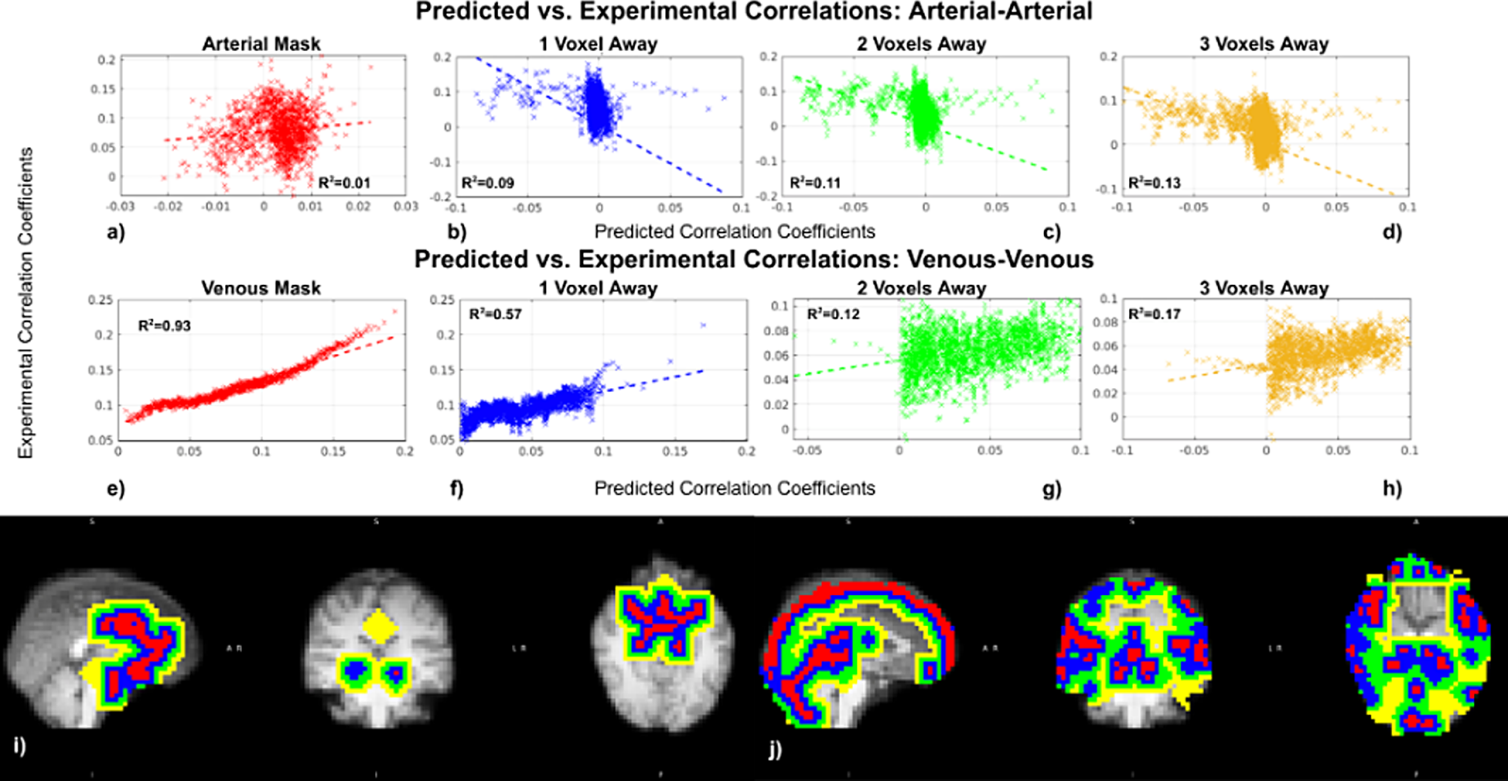
Dependence of perivascular correlation coefficients on distance fromvasculature: predicted versus experimental results (macro-VANModel). Data were taken from the same representative subject asshown inFigure 7. Anillustration of arterial–arterial correlation (a–d)and venous–venous correlation (e–h). Perivascularmasks are shown for both arterial and venous, with colour coding asred—intravascular, blue—one voxel away from thevascular voxel, green—two voxels away, andyellow—three voxels away (i, j). Each dot represents a binaverage.

Unlike for venous RSFA, venous-driven correlation remains quite wellpredicted by the macro-VAN Model at one voxel away (voxels are 4 mmisotropic) from the vascular voxel, as shown by the R^2^value(Fig. 10f). The regression remainssignificant at two and three voxels away, however, R^2^decreasedsubstantially (Fig. 10g, h). Thepredictability of arterial-driven correlations remains poor for allperivascular distances (Fig.10a–d). SeeFigures S7 and S8in Supplementary Materials forresults for all subjects.

### 3.5 Comparison of three models across all subjects

**Fig. 11.**
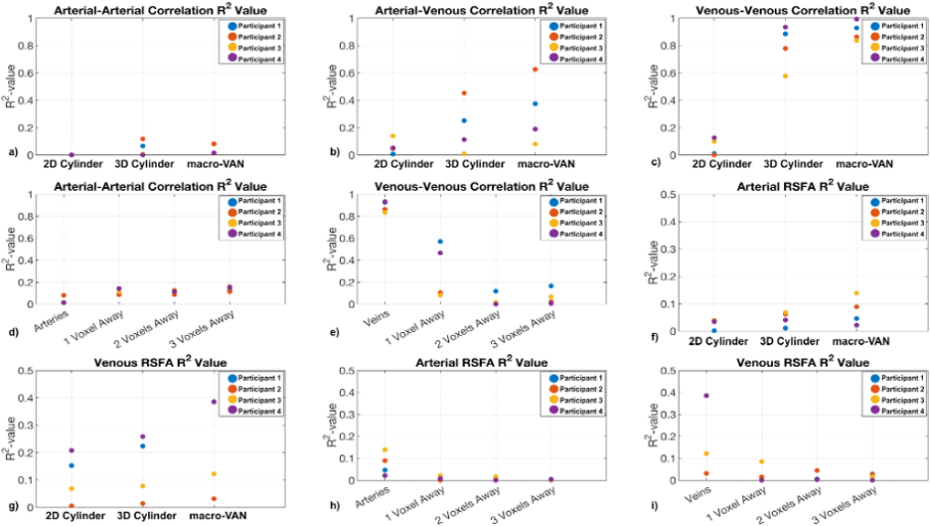
Summary of results from all subjects: the agreement of predicted andexperimental FC driven by vasculature, quantified by R^2^.R^2^values are plotted for all four data sets for (a)arterial–arterial correlation, (b) arterial–venouscorrelation, (c) venous–venous correlation, (d)arterial–arterial perivascular correlation, (e)venous–venous perivascular correlation, (f) arterial standarddeviation, (g) venous standard deviation, (h) arterial perivascularstandard deviation, and (i) venous perivascular standard deviation.

Comparisons of FC-prediction R^2^values from the three models based ondata from all four subjects are summarized inFigure 11. In general, the macro-VAN Model provided the highest FCprediction accuracy (i.e., R^2^values), followed by the 3D CylinderModel, and lastly the 2D Cylinder Model (Fig.11a–c). Additionally, the simulated venous–venous FCvalues (Fig. 11c) are associated with thehighest R^2^values (all <0.8), followed byarterial–venous FC values (Fig.11b), and then arterial–arterial FC values (Fig. 11a). Moreover, perivascular prediction differencesare also summarized inFigure 11acrossall subjects and distances. Generally, the R^2^values ofarterial–arterial FC were low, and increased with distance away from theoriginal vascular mask (Fig. 11e). As withthe FC simulation, the RSFA simulation shows a similar pattern, but withgenerally lower R^2^. Both arterial and venous signals were betterpredicted by the macro-VAN Model than by the other models (Fig. 11f, g). Moreover, there is a trend of decreasinggoodness of fit with increasing distance from the macrovascular voxel (Fig. 11h, i).

## 4 Discussion

Despite the strong influence of macrovascular contributions to BOLD fMRI (Huck et al., 2023;Menon, 2012;Tong et al.,2015;X. Z. Zhong et al., 2024),previous research demonstrated that removing this bias is not an easy task (Huck et al., 2023;Menon, 2002;Ragot& Chen, 2019;Stanley et al.,2021). Using biophysical simulations informed by angiography data, thepresent study examines the feasibility of predicting the macrovascular BOLDcontributions proximal and distal to the vasculature. We explored the use of awhole-brain macro-VAN Model as well as single infinite-cylinder approximations (2Dand 3D). We found that:

1. With the availability of angiographic data, it is feasible to modelmacrovascular BOLD FC using both the macro-VAN-based model and 3Dinfinite-cylinder models, though the former performed better.
2. Biophysical modelling can accurately predict the BOLD FC (in terms ofcorrelations) for veins, but not for arteries.
3. Biophysical modelling provided less accurate predictions for RSFA than forFC.
4. Modelling of perivascular BOLD connectivity was feasible at distances closeto veins but not arteries, with performance deteriorating with increasingdistance.

### 4.1 Origins of the resting-state macrovascular BOLD fluctuations

The macrovascular contribution to the rs-fMRI BOLD signal has many physiologicalorigins. The concept of systematic low-frequency oscillations (sLFOs) was firstproposed in an earlier review article and defined as a low-frequency vasogenicBOLD signal travelling through the brain (Tonget al., 2015). This phenomenon does not appear to originate withinthe brain (Frederick & Tong, 2010;Li et al., 2018;Tong & Frederick, 2010) and is likelyassociated with heart rate variability (Thayeret al., 2012), gastric oscillations (Mohamed Yacin et al., 2011;Rebolloet al., 2018), vasomotion (Hundley etal., 1988;Mayhew et al.,1996;Rivadulla et al., 2011),respiratory volume variability (Birn et al.,2006;Chang et al., 2009),and/or variations in carbon dioxide levels (Sassaroli et al., 2012;Wise et al.,2004), among others. In previous studies using near-infraredspectroscopy, it was demonstrated that such oscillations travel through theperipheral to the cerebral vasculature (Frederick et al., 2013;Tong& Frederick, 2012,^2014^;Tong, Bergethon, et al.,2011;Tong, Hocke, et al.,2011;Tong, Lindsey, et al.,2011;Tong et al., 2014,^2015^). As venous blood is predominantlyresponsible for the BOLD signal (Ogawa, Lee, etal., 1993;Segebarth et al.,1994), the presence of sLFOs may suggest that certain findings fromrs-fMRI experiments may not reflect neural activity but rather venous bias(Aso et al., 2020).

To understand the influence of the macrovasculature on rs-fMRI, it is essentialto have a basic biophysical understanding of the macrovascular BOLD effect. Themacrovascular contribution is likely to be strongest within the confines of bothmacrovascular and perivascular tissues, as discussed in our previous study(X. Z. Zhong et al., 2024), but afull understanding of the large variability in the perivascular BOLD effectacross brain regions and acquisitions (Huck etal., 2023;X. Z. Zhong et al.,2024) cannot be obtained from in-vivo experiments alone. We hope thatour simulated results can contribute to a better understanding of thisphenomenon.

### 4.2 Comparison of simulation models

#### 4.2.1 Theoretical comparisons of our models

The three simulation models compared in this study range in complexity andrealism from low to high, namely from the 2D infinite-cylinder Model, to the3D infinite-cylinder Model, to the empirical macro-VAN Model. Unlikemicrovascular BOLD simulations (Berman etal., 2018), macrovascular simulations cannot ignore fBV changescaused by vessel obliquity, as macrovascular fBV is more substantial andarises from much larger “cylinders.” The 3D Cylinder Model isexpected to be more accurate than the 2D Cylinder Model due to its abilityto capture the effect of orientations (azimuth and zenith) on fBV. As anexample, a vessel with an azimuth angle of 45° or zenith angle of45° would have an fBV of approximately 1.41 times that of a vesselwith both angles at 0. Thus, the 2D Cylinder Model is expected to beoutperformed by the 3D Cylinder Model, which also models vessels ascylinders but more accurately captures fBV and vessel orientation. While the2D Cylinder Model was expected to provide low accuracy, it was unclear apriori how these simplifying assumptions would affect our BOLD predictions,and given its practical advantages it was evaluated in our study. Ourconclusion is that, for modelling large vessels, the 2D model is inadequateand the disadvantages outweigh the advantages.

Likewise, we expected the macro-VAN Model to produce the most realistic BOLDsimulations since it incorporates the shape and position effects of realmacrovasculature that cannot be captured by the 2D and 3D Cylinder Models.The macro-VAN Model can capture details missing from the 3D Cylinder Model,such as the geometry of blood vessels (with bends, branching, etc.), partialvolume effects, and positional effects (when the vessel is not centred in avoxel). Despite the advantages of the macro-VAN Model, it still has somelimitations. Because it is based on the Fourier-based discreteGreen’s function, the accuracy of this approach depends on the gridresolution. More specifically, to ensure accuracy, the diameter of thesmallest blood vessels should span at least four voxels (Tang et al., 1993). Coarse spatial sampling ofthe vascular susceptibility gradient and boundaries leads to truncation or“ringing” effects, which can be alleviated by spatiallyupsampling, thus smoothing of the kernel function. This is in contrast tothe 2D Cylinder Model, which can be constructed at an arbitrarily highspatial resolution (by using smaller sub-voxels) to provide a more accuraterepresentation of fBV variations, which is especially relevant for smallfluctuations in the resting state. Furthermore, given the complexity of themacro-VAN Model, a more intuitive understanding of the BOLD signaldependence on vascular geometry is challenging. Such an understanding can bemore easily achieved with the 2D Cylinder Model, which allows for the effectof different geometric parameters to be examined in isolation (Ogawa, Menon, et al., 1993).Furthermore, the computational complexity of the macro-VAN Model is also thehighest, as it takes a considerable amount of computational memory andprocessing time to simulate the entire macrovasculature, as shown in thenext section. The 3D Cylinder Model provides a trade-off between geometricalaccuracy and computational requirements, and was investigated as a morepractical alternative to the macro-VAN Model, to potentially make suchmodelling methods more practical.

#### 4.2.2 Model computational requirements

We also note that high accuracy comes at a cost. The macro-VAN Model canconsume as much as 160 GB of memory and 75 h of computation time forarterial simulations alone. The 3D Cylinder Model, though much simpler (2 GBmemory and 108 h computation time), remains much more computationallydemanding than the 2D Cylinder Model (1 GB memory and 7.5 h computationtime) (Table 3). According to thediscussion above, the 2D Cylinder Model could perform very similarly to themacro-VAN Model in certain scenarios, and in fact, mostS_v,model3_:S_v,model1_values are lower than 3(zoomed window ofFig.S1bin Supplementary Materials). It is the same for theprediction of correlation coefficients. Thus, in order to select theappropriate simulation model, it is important to consider the errortolerance and the availability of computational resources. Our study is notfocused on numerical algorithms, and the computational resources listed hereare only meant to provide a comparison and may not be based on the mostefficient algorithms or reflect all hardware architectures.

**Table 3.** Computational resource for each model(h = hours).

	Computational memory (GB)	Computational time (h) per participant
macro-VAN Model	160	96 (arteries), 2.5 (veins)
2D Cylinder Model	1	1.5 (arteries), 7.5 (veins)
3D Cylinder Model	2	60 (arteries), 108 (veins)

### 4.3 Theoretical understanding of macrovascular BOLD

According to the classic biophysical model proposed byOgawa, Menon, et al. (1993), the BOLD signal isdependent on the vascular orientation, size, and oxygenation. θ and fBVare two parameters widely investigated in relation to BOLD signal dependencies(Ogawa, Menon, et al., 1993;Viessmann et al., 2019;X. Zhong & Chen, 2022). The ΔBOLDmetric, calculated based on the difference in the BOLD signal corresponding tothe two simplified cases of the input signals (variations in fBV for arteriesand in Y for veins), was used to demonstrate the dependency of each parameter onthe simulated BOLD fluctuations, whose magnitude should be proportional to theRSFA and whose polarity will directly affect the correlation with other BOLDsignals from other locations.

In contrast with previous experimental observations at 7 T based primarily onmesoscopic pial veins and the associated extravascular fields (Viessmann et al., 2019), the magnitude ofmacrovascular ΔBOLD in this work does not consistently decrease withincreasing θ, but is rather minimized at θ ~ 3π/4 (Fig. 6m, n), which, according to the originalbiophysical model (Ogawa, Menon, et al.,1993), leads to zero intravascular magnetic field offset. Themacrovasculature, in contrast to microvasculature, has a higher fraction ofintravascular volume, such that its IV contribution cannot be neglected. It isintriguing to note that, although venous ΔBOLD remains positive for allfBV, arterial ΔBOLD changes polarity from negative to positive with anfBV of ~0.4 (Fig. 6o, p). It is possiblethat a decrease in the extravascular compartment associated with an fBV increasecould lead to a dominance of intravascular contribution to the measured BOLDfluctuations. (In the case of arteries, an increase in the intravascularcompartment would be expected to increase phase homogeneity, since allintravascular spins in a given voxel would be in phase.) As a result of thechange in polarity of the simulated signal, arterial–arterialcorrelations could potentially be negative with specific sets of fBV, just asthe arterial–venous correlation could be positive. These complicateddependencies suggest that multiple factors must be considered simultaneouslywhen modelling macrovascular contribution to both BOLD fluctuation magnitude andcorrelation, and that simply considering diameter would not suffice forvoxel-based modelling (Huck et al.,2023).

However, these are not the only parameters that could affect the simulated BOLDsignals. Our previous study demonstrated that even unexpected parameters such asthe position of a vessel within a voxel can have an impact on macrovascular BOLD(Sedlacik et al., 2007;X. Zhong & Chen, 2023) and this canbe extended to the impact of partial-volume effects. It is, however, difficultto measure the exact position of vessels within the fMRI voxels in our in-vivoexperimental data, so the simulation was not able to include this effect. It isalso possible that our results may be affected by other parameters (such as TE,spatial resolution, and the main magnetic field), which are not addressed inthis initial study but could be considered in the future.

### 4.4 Experimentally measured macrovascular BOLD: agreement with prediction

#### 4.4.1 Model prediction of macrovascular BOLD fluctuation amplitudes

A general conclusion can be drawn that the macro-VAN Model provides betterpredictions than the 2D Cylinder and 3D Cylinder Models for RSFA (Figs. 7and11). Interestingly, the macro-VAN Model was able topredict venous-driven RSFA reasonably well. As discussed in the theorysection, the macro-VAN Model has the most realistic geometric representationof macrovasculature, and while the 3D Cylinder Model is less realistic, itstill embodies the interactions between vascular orientation and fBV,whereas the 2D Cylinder Model does not. This is also reflected in thecomparison of the simulated baseline BOLD signal intensities (seeFig. S1inSupplementary Materials), whereby the unrealistic geometry of the 2DCylinder Model leads to predicted signal fluctuation amplitudes that deviate(up to 60%) from that of the macro-VAN Model. Nevertheless, none of thethree models is capable of providing satisfactory prediction for arterialRSFA. In the 2D Cylinder and the 3D Cylinder Models, we observed a clusterof low simulated values that do not fit well into a linear relationship,which suggests unreliable prediction for some of the voxels.

Moreover, the RSFA prediction accuracy is highly participant dependent asshown inFigure 11, which may in turnbe dependent on the parameters of the acquisition and on the distinctanatomical structures of each participant (e.g., participant positioning andgeometry of the macrovasculature). Moreover, different participants may alsobe affected by different levels of physiological noise (e.g., heart ratevariability, respiratory variability), which further increases the level ofinter-subject variability. As well, the effect of motion artefacts cannot beignored. Different participants may have different levels of motion (eithergross head motion or local physiological pulsation) which result indifferent levels of noise for both rs-fMRI and TOF imaging.

In general, unlike for venous RSFA, none of our models provided satisfactorypredictions of arterial RSFA in any of our subjects (Fig. 11). Since the diameter of the arteries isconstantly changing, it is almost impossible to establish an accuratebaseline diameter for use in our simulations. Additionally, the duration ofthe TOF angiogram acquisition is significantly longer than the arterialvariation (the MRA acquisition in the MSC data set lasted approximately 5min) (Gordon et al., 2017;Reimers et al., 2018), resulting in afurther blurring of the arterial vasculature boundaries. A further challengeis emulating arterial movement in simulation, particularly for the macro-VANModel, which requires extremely high resolution and is, therefore, notpracticable. While some state-of-the-art acquisition techniques allowdynamic imaging of arteries, they still only cover one blood vessel ratherthan the entire vasculature (Varadarajan etal., 2023). Further research to improve the temporal resolutionof angiograms will be necessary.

#### 4.4.2 Model prediction of macrovascular FC

The discrepancy between the predicted and the experimental FC could be, inpart, due to the simulated signal having a much higher signal-to-noise ratio(SNR) than the in-vivo signal. To account for this, we weight the simulatedconnectivity with the simulated RSFA in order to emulate the in-vivoexperimental signals better (Golestani& Goodyear, 2011), as described in the methods section.This approach prevented the FC maps from being dominated by largevessels.

Like in the case of the RSFA, the macro-VAN Model resulted in higher FCprediction accuracy than the 2D and 3D Cylinder Model (includingarterial–arterial, arterial–venous, and venous–venousFC). The 3D Cylinder Model in turn performed better than the 2D CylinderModel in all cases (Fig. 7).Venous–venous correlation was remarkably well predicted by themacro-VAN Model, followed by arterial–venous correlation. Both themacro-VAN Model and the 3D Cylinder Model generated satisfactory predictionsfor venous–venous correlation, which suggests that the 3D CylinderModel may be used as a substitute for the macro-VAN Model. However, for the2D Cylinder Model, we noted in cases with poor prediction that the simulatedvalues appear to be sub-divided into two groups, the first spanningcorrelation coefficients greater than 0.04 and the second spanningcorrelation coefficients between 0 and 0.04. The existence of two clustersof correlation coefficients may result from signal intensity outliers asdiscussed above (see*Model Theoretical Comparison*). Inlight of the results shown inFigure S1in Supplementary Materials, we suggest thatthe 2D Cylinder Model is likely to produce spurious BOLD signal fluctuationamplitudes with specific vascular orientation and fBV values that maydistort the predicted time courses, which translate into extreme RSFA valuesand thus widely fluctuating FC values.

Similar to the case of RSFA, we were not able to predictarterial–arterial correlation with any of the models. Interestingly,predicted arterial–venous FC values were anti-correlated with theexperimental values (Fig. 8d–f).Arterial BOLD contributions are generally weaker than venous contributions,whereas the angiograms are more sensitive to the fast-flowing arterial bloodthan the slower moving blood of the neighbouring veins (such as thecavernous sinus, which is in proximity to the Circle of Willis). Thus, whena single voxel contains both an artery and a vein, one dominating thesegmentation and the other dominating the susceptibility effect, the modelprediction may be particularly challenging. Certainly, given the pooraccuracy of arterial predictions seen in this study, it is clearly morefeasible to pivot towards estimating large-vein contributions using ourapproach.

Our R^2^values are lower than those of the binned predictionsreported byHuck et al. (2023)(R^2^> 0.98), but much higher than those of theirvoxel-wise predictions (R^2^< 0.05). The results of ourstudy, although still based on binning, are based only on predictedcorrelation coefficients from first principles, which should allow betterrepresentation of the voxel-wise macrovascular effects. SinceHuck et al. (2023)also examined otherconnectivity metrics (low-frequency fluctuation amplitude, regionalconnectivity inhomogeneity, Hurst exponent, and eigenvector centrality), adirect comparison of these two studies is difficult.

Interestingly, our FC predictions appear to be more accurate than ourprediction of RSFA, especially when comparing venous RSFA andvenous–venous correlation. There may be several reasons for this.First, measured RSFA likely includes factors that are not incorporated inour model such as multiplicative scaling from receive coil sensitivities,which may scale amplitudes of the fluctuations but not their timing.Correlation coefficients are normalized quantities and thus are lessinfluenced by these regional scalings. Second, following similar logic, theRSFA and correlation coefficient are also differentially sensitive topartial-volume effects. Third, factors such as vascular position in a voxelalso affect signal intensity fluctuations but not necessarily thecorrelation (X. Zhong & Chen,2023). The basic concept stems from the fact that a given voxelmay not be able to sample the entire dipole, but the details are beyond thescope of this paper. The same argument can be extended to the relativeimmunity of correlation coefficients to errors in estimating fBV andorientation from the segmented macrovasculature because of how they arenormalized. Such errors will also have a greater impact on the fluctuationmagnitude than the temporal features of the fluctuations.

#### 4.4.3 Model prediction of perivascular BOLD effects

In both arteries and veins, our model was unable to predict the in-vivoexperimental RSFA at locations farther than one voxel away (~4 mm) from themacrovasculature. This may correspond to different number of voxelsdepending on the voxel size. A potential explanation for the unsatisfactorypredictions could be found in the RSFA maps (Fig. S6inSupplementary Materials), which suggest that the macrovascularsusceptibility effect may only extend, or be predictable, over a limiteddistance from the macrovasculature. Consequently, in ROIs with limitedmacrovascular susceptibility effects, the in-vivo signal may be dominated byother sources of fluctuations (e.g., neural activity and physiologicalnoise), which cannot be reflected by the predicted RSFA.

From the perspective of correlations, we found that in-vivovenous–venous correlations, while extremely well predicted by themacro-VAN Model, also became substantially less well predicted at one voxelaway from the macrovasculature. This finding is similar to that of aprevious study that found that the contribution of the veins to connectivitydecreased with distance from the venous vasculature (Huck et al., 2023). Arterial–arterialcorrelations were not well predicted by the simulations even in the voxelscontaining the vessels.

One of the important questions raised by our previous studies is whetherperivascular FC is related to EV magnetic susceptibility or to contributionsof undetected vessels. As discussed in the previous section, the magneticsusceptibility difference between blood and tissue would generate anextravenous dipolar magnetic field offset around the macrovasculature thatcould extend well beyond the voxel containing the vessel (Ogawa, Menon, et al., 1993). We observed thesusceptibility effect become attenuated rapidly beyond the macrovascularvoxel (Fig. S6inSupplementary Materials), unlikely to persist more than 4 mm away from themacrovasculature (even when thresholded a 1% of the maximum RSFA). Ourregression results, for both RSFA and FC, support this claim. Such would bethe case whether the macrovascular R_2_’ perturbations aredue to neuronal activity or other physiological processes. Of course, somephysiological noise effects are global (Birnet al., 2006;Chang et al.,2009;Tong et al., 2015);however, they would not cause the model performance to deteriorate withincreasing distance from the macrovasculature. Since the latter is what wesee, we propose that EV susceptibility effects are not the sole contributorto perivascular BOLD. For one thing, there are numerous medium-to-smallblood vessels in the extra-macrovascular space that are not detected by theTOF acquisition due to their smaller size and lower flow velocity. Theirunmodelled BOLD contributions, which are likely also modulating the BOLDsignal behaviour more distal to the microvasculature, may be the main reasonthat our modelling accuracy deteriorates with increasing distance from themacrovasculature. Our future work will target the improvement ofmacrovascular detection sensitivity, perhaps through the use of contrastagents (Bernier et al., 2020).

### 4.5 Recommendations

This study aims to demonstrate that modelling the macrovascular BOLD FC (in termsof correlation coefficients) using a biophysical model is feasible, and canprovide a post-acquisition means to reduce the macrovascular bias during dataanalysis and interpretation in rs-fMRI. This study demonstrates, in concept,that TOF data acquired within rs-fMRI sessions can be used to predict themacrovascular bias, as we suggested in our previous study (X. Z. Zhong et al., 2024). The use of a fullmacrovascular VAN (the macro-VAN Model in this work) through numericalfield-offset calculations demonstrated the highest performance in predictingvenous-driven macrovascular FC, and thus should be the starting point for futureefforts to eliminate macrovascular contribution on FC. To address the highcomputational cost of such an approach, pre-computed look-up tables could bedeveloped that encapsulate all possible simulated signals, thereby increasingthe accessibility of our approach. However, the prediction of arterialmacrovascular BOLD is poor, potentially calling for better methods of capturingarterial dynamics. Lastly, perivascular BOLD contributions by large veins couldbe modelled reasonably accurately at ~4 mm beyond the vascular voxel, but notmore. This could speak of the need to use more sensitive TOF imaging and higherspatial resolution to model perivascular effects from smaller vessels.

Based on the current results, adjusting FC values based on simulated FC would bethe most promising way to reduce the macrovascular effect on resting-state fMRI.That is, macrovascular contributions to FC could be accurately predicted by ourmacro-VAN Model. It is, however, important to realize that such an FC correctionis currently only realizable on the cross-correlation coefficients (instead ofPearson’s correlation, e.g.) between voxels in the vicinity of largeveins. This is because our models do not account for lags between times seriesand their effects on correlation coefficients. Besides correlations, there mayalso be other, lag-insensitive approaches for calculating FC, such as regressionand independent component analysis (ICA), but adapting our investigation tothese other metrics will be the subject of our future work.

### 4.6 Study limitations

This study has several limitations. This report will not discuss the limitationsof in-vivo experiments, which are discussed in our previous work (X. Z. Zhong et al., 2024). We will insteadfocus on the limitations most relevant to the simulations.

First, the comprehensiveness of the simulations is limited by the sensitivity ofthe TOF. Thus, we are unable to model the entire macrovasculature, which makesthe input macrovasculature imperfect for fully accurate simulations and willreduce prediction performance. Moreover, the accuracy of vascular fBV estimatescan be biased by differences in flow rates across vessels. Additionally, thesimulation is based on simple sinusoidal signals as inputs to mimicresting-state fluctuations, which is likely not realistic (for either fBV or Y).Also, certain parameters, particularly for the 2D and 3D Cylinder Models, wereshown to be important but were difficult to extract from TOF images, includingthe position of the vessel within the fMRI voxel (X. Zhong & Chen, 2023). Moreover, there havebeen various definitions of “macrovasculature” in the literature.Recognizing that not all venous BOLD effects are artefactual, we will adhere tothe definition of “macrovasculature” outlined inMenon (2012)In our specific case,“macrovascular” refers to vessels detectable from our TOF MRAdata, which are far larger in diameter than the intracortical veins.Furthermore, the TOF preprocessing procedures, such as brain extraction, mayhave led to some loss of macrovascular voxels, such as at the periphery of theprefrontal cortex. Future analysis will target ways to maximize vascularsegmentation completeness using improved tools.

Secondly, due to the nature of TOF contrast, it is impossible to rely on thedirectionality of flow to distinguish between arteries and veins. Moreover, dueto the proximity of some arteries and veins (such as the Circle of Willis andthe Cavernous Sinus), partial-volume effects cannot be completely avoided. Thislimitation is mentioned in the Limitations section. While this adds to thechallenge in identifying macrovasculature, we wish to point out that (1) themajority of venous data points are from identifiable veins such as the sagittalsinus and transverse sinus; (2) the venous BOLD effect is much stronger than thearterial BOLD effect, likely contributing to the high predictability from“venous voxels” compared with arterial ones in this work despitepotential spatial overlaps between arteries and veins in some regions.

Thirdly, the availability of computational resources also limits the feasibilityof the simulation. In spite of having more than 180 GB of memory, our server canonly support 0.2-mm resolution for the macro-VAN Model simulation, which is notsufficient for some narrow segments of the macrovasculature. The longcomputational times for the simulations are another factor, which only allowedus to include four participants within a reasonable time frame. There is apossibility that this problem may be alleviated by the addition of computationalresources in the near future.

Lastly, the applicability of these results likely depends on scan parameters(including spatial resolution, main magnetic field, and temporal resolution) ofthe MSC data set; therefore, comparisons with previous studies, especially thosewith higher main magnetic fields, should be made with caution. In practice,predictions should be tailored to different scanning protocols.

## 5 Conclusions

To conclude, we found that the macrovascular FC could be predicted using biophysicalmodelling based on vascular anatomical information, and that the venouscontributions could be more accurately predicted than the arterial contributions. Inaddition, we found that the models were less reliable in predicting macrovascularRSFA than FC. Moreover, we found that modelling perivascular BOLD effects usingsimulations is feasible at a distance close to macrovasculature, also moreaccurately for veins than for arteries. This study paves a path for model-basedcorrection of the macrovascular bias in resting-state and other types of fMRI, thevalidation of which will be part of our future work.

## Supplementary Material

Supplementary Material

## Data Availability

All data described in this manuscript were obtained from the Midnight Scan Club (MSC)data set. The data are available publicly at OpenNeuro. The code will be madeavailable upon request.
